# Treatment limitations and participation in elderly patients – the gap between medical-ethical guidelines and clinical practice: a cross sectional-study from Sweden

**DOI:** 10.1186/s12877-025-06552-x

**Published:** 2025-11-04

**Authors:** Fredrik Hessulf, Matts Juhlin-Dannfelt, Björn Agvall, Anders Bremer, Viveka Andersson

**Affiliations:** 1 Department of Anaesthesiology and Intensive Care Medicine, Sahlgrenska University Hospital, Mölndal, Sweden; 2 Department of Anaesthesiology and Intensive Care Medicine, Institute of Clinical Sciences, Sahlgrenska Academy, University of Gothenburg, Gothenburg, Sweden; 3 Andersberg Primary Healthcare Centre, Region Halland, Halmstad, Sweden; 4 Department of Research Development, Region Halland, Halmstad, Sweden; 5 Center for Primary Health Care Research, Department of Clinical Sciences, Lund University, Malmö, Sweden; 6 Department of Health and Caring Sciences, Faculty of Health and Life Sciences, Linnaeus University, Växjö, Sweden; 7 Department of Rehabilitation/Medicine, Halland Hospital Varberg, Varberg, Sweden

**Keywords:** Treatment limitation, Aged, Cardiopulmonary resuscitation, Resuscitation orders, Frailty, Comorbidity, Ethics

## Abstract

**Background:**

Decision-making regarding treatment limitations such as “Do not attempt resuscitation” (DNAR) orders for older patients has been found deficient. Patients ≥ 80 years with substantial comorbidity have little chance of surviving cardiac arrest, thus require thorough risk classification focusing on comorbidity and frailty. This study aimed to explore the degree of frailty, comorbidity and treatment limitations in patients ≥ 80 years in various forms of care. Additionally, the study examined the extent to which patients and/or relatives participated in these decisions.

**Methods:**

Descriptive, quantitative cross-sectional design. Medical records of 500 patients ≥ 80 years were reviewed: 100 medical, 100 orthopaedic and 100 surgical in-patients, in addition to 100 patients in Home Health Services (HHS) and 100 patients in Municipal Short-Term Care (MSTC). Comorbidity was classified and categorized using the Age-combined Charlson Comorbidity Index (ACCI). Frailty was assessed using the Clinical Frailty Scale (CFS). DNAR decisions as well as other treatment and care limitations were compiled. Patients’ and relatives’ participation in discussions and information about treatment limitations was also examined.

**Results:**

Of the 500 patients, 48% had a moderate (5–7 points) and 50% a severe burden (≥ 8 points) of ACCI, while 91% were rated as frail (CFS ≥ 5). In total, 176/500 (35%) had valid DNAR-decisions. Both age ≥ 90 years (OR 4.07, 95% CI 2.56–6.37) and CFS ≥ 5 (OR 16.13, 95% CI 4.54–103.40) was significantly associated with a DNAR-decision, while ACCI ≥ 8 was not. Less than a third (29%) of patients with a DNAR-decision had been informed by a physician. For those without a DNAR-decision, there was no documentation of discussions regarding their wish for full cardiopulmonary resuscitation (CPR) in the event of cardiac arrest. Of all 500 patients, 14% had a discussion with a physician about CPR.

**Conclusion:**

Fewer treatment limitations than expected were documented for older, frail patients with moderate or severe comorbidity. Considerable deficiencies were found regarding decision-making and actively reviewing and confirming DNAR-decisions, showing a gap between medical-ethical guidelines and their application in practice. Improved adherence to medical-ethical guidelines would strengthen patients’ legal rights and their opportunity for shared decision-making.

**Supplementary Information:**

The online version contains supplementary material available at 10.1186/s12877-025-06552-x.

## Background

Current practice regarding treatment limitations, such as “Do not attempt resuscitation” (DNAR) for older patients cared for in hospital and other healthcare settings, has been found deficient [1]– [2]. Despite technical developments and guidelines for the application of DNAR orders, survival rates following cardiac arrest are less than 10% in patients ≥ 80 years with substantial comorbidity [3–5]. Survival is often followed by poorer health status, intensive care and cognitive decline. A balance between ethical values, such as doing no harm, doing good, equality of care, human integrity and autonomy should guide decisions about treatment limitations [6].

Patients ≥ 80 years require careful risk classification. The burden of comorbidity, combined with functional status, older age and type of cardiac arrest setting, is associated with the likelihood of neurologically intact survival [7]. After cardiopulmonary resuscitation (CPR), 30-day survival among nursing home residents is 1.7% and 4.9% for those in home care [8]. For patients suffering cardiac arrest in hospital, survival is higher [7]. Swedish hospital register data showed that 30-day survival after cardiac arrest was 20% for those aged 80–89 years and 14% for those ≥ 90 years, while patients with significant comorbidity, organ dysfunction or cancer had a much poorer outcome [4]. A study using the Age-combined Charlson Comorbidity Index (ACCI) revealed that those aged ≥ 80 years with moderate or severe comorbidity had a small chance of surviving a cardiac arrest [3].

Documentation of treatment limitations aimed at protecting frail, older and terminally ill patients from futile and stressful interventions is an integral part of modern medicine [6]. The European Resuscitation Council Ethics Guidelines underline the patient’s right to a discussion about, and participation in, CPR-decisions [9]. However, the implementation and documentation of DNAR-decisions face several barriers. Patients and relatives may overestimate the chance of survival [10]. Barriers for staff may concern lack of experience, fear of making mistakes, time constraints, lack of senior staff and clear guidelines [10]– [11]. Physicians can be uncomfortable discussing DNAR orders with patients and their families [12]. However, studies have demonstrated that patients may find it valuable to discuss treatment limitation issues [13].

The Swedish National Board of Health and Welfare as well as European CPR guidelines emphasize that decisions on treatment limitations should follow the ethical principles of autonomy, beneficence, non-maleficence and justice [14]– [15]. In Sweden, ethical CPR guidelines allow a DNAR-decision based on the following criteria: patient does not want CPR, or physicians consider it futile or of no benefit to the patient. No benefit refers to situations where the potential benefits of CPR are outweighed by the risk of severe complications that could impair the patient’s quality of life, physical function or neurological status. To be considered valid, decisions to withhold CPR should be reviewed regularly as well as confirmed/reviewed at transfer to another care unit [16].

Swedish guidelines recommend documentation of discussions with patients regarding CPR-decisions or, if not feasible, that relatives were informed and how they received the information [14]. Patients should be offered information about their health, treatment options and potential risks, when appropriate involving relatives [17]. Studies indicate that these recommendations were fully met in only 10% of cases, with patient participation documented in just 28%, mainly due to cognitive impairment, diminished consciousness or acute situations during DNAR decision-making [18]– [19].

Frailty is a multi-dimensional syndrome characterised by increased vulnerability to adverse health events [20] and a robust predictor of how older patients cope with such events [21]– [22]. Its presence is associated with a threefold risk of death after cardiac arrest [23]. Identifying “Goals of care” involves the patient’s expressed values and the aim of the planned treatment [24]– [25]. Age, frailty and comorbidity are essential components in this process [25].

Studies focusing on the extent to which decisions about treatment limitations are made for older patients with comorbidity and frailty across different care settings are needed. This complex area encompasses both medical and ethical dimensions. The aim of this study was to explore the degree of frailty and comorbidity in documented decisions regarding CPR and other treatment limitations in older adults receiving different types of care. Additionally, the extent to which patients and/or their relatives participated in these decisions was investigated.

## Method

### Study design and setting

This study utilised a descriptive, quantitative cross-sectional design. Data were gathered by reviewing electronic medical records from hospital settings, Municipal Short-term Care (MSTC) and Home Health Services (HHS), focusing on individuals ≥ 80 years. The study was conducted in a region located in southwestern Sweden, with a population of about 340,000. The healthcare infrastructure in the region includes two emergency hospitals and 25 inpatient wards. The healthcare system also encompasses MSTC, which offers temporary residential care for patients needing short-term rehabilitation, recovery or medical attention. Additionally, HHS in the region provide medical and nursing care in patients’ private residences and nursing homes. This healthcare structure supports individuals with chronic conditions and cancer through coordination between municipal nurses and primary care physicians.

There were no standardised DNAR protocols in any of the units. Physicians actively marked a decision under an alert symbol in the electronic medical record when a DNAR decision is made. The decision taken should also be documented in free text in the medical record. The following information should be included: the reasons for the decision, whether the patient has been informed, how the patient perceives the information/decision and whether relatives have been informed [14, 17]. Confirmed or reviewed decisions, for instance when a patient is transferred to another unit, must also be signed under the alert symbol with the current date [16]. At the units studied, regular practical training in CPR is offered to both physicians and nurses. However, there is no organised education regarding the national ethical guidelines for CPR [16].

### Data collection

Reviews of medical records were conducted for 500 patients ≥ 80 years. Three hospital care settings were included: medical, orthopaedic and surgical, in addition to 100 patients from MSTC and HHS, respectively. The medical records of patients admitted for at least 24 h at the time of the study were examined in the order in which they appeared in the patient register at the respective unit. Patients were only included once. The review took place one day per week over a 12-month period, between May 2023 and April 2024.

A two-part, study-specific form was employed for data collection. Part one included demographic and clinical information; age, gender, frailty level, current diagnosis and comorbidity, care duration and time from admission to treatment limitation decision. Part two addressed specific treatment limitations and participation in decision-making by patients and/or relatives tailored to each care setting (hospital ward, MSTC or HHS).

### Outcome measures

DNAR decisions were classified as valid if they were made and documented during the current episode of care. Decisions made during a previous episode or in another form of care were also considered valid if they had been actively confirmed by a physician during the current episode. Older decisions from previous care settings that had not been confirmed during the current episode were classified as non-valid. The classification was made in accordance with the Swedish ethical guidelines for CPR [16]. Both valid and non-valid treatment limitations were included in the analysis to reflect real-world data.

Patient involvement was reviewed in medical record entries documented in connection with the DNAR decision. Information provided to relatives was also reviewed in the medical record. According to Swedish legislation, verbal information provided to the patient regarding treatment limitations and, if applicable, to relatives should be documented in the patient’s medical record [14, 17].

Comorbidity was evaluated by recording patients’ chronic diagnoses in accordance with the International Classification of Disease-10 (ICD-10) [26]. In addition, the ACCI [27]– [28] which assigns severity-weighted points for age and chronic health conditions associated with 1-year survival was used. Patients’ frailty level was measured in accordance with the Clinical Frailty Scale (CFS) [20, 29]. Records comprising patients’ status as documented by physicians, nurses and physiotherapists were scrutinised and evaluated, followed by categorisation of each patient from 1 to 9 on the CFS; 1–3 (non-frail), 4 (vulnerable or pre frail), 5 (mildly frail), 6 (moderately frail), 7 (severely frail), 8 (very severely frail) and 9 (terminally ill). Two researchers (a physician and a nurse) conducted the review. Thus, frailty was uniformly evaluated with consensus between them.

### Statistical analysis

Data are summarised using descriptive statistics. Categorical variables are reported using counts and percentages. Continuous variables are presented as means with standard deviations. Comparison between groups were conducted using the Chi-square test for categorical variables and ANOVA for continuous variables. Age was categorised as 80–89 years or ≥ 90 years. The frailty score was categorised as 1–4 (non-frail) and ≥ 5 points (frail). In the ACCI, age-combined co-morbidities were categorised as 0–4 (low burden), 5–7 (moderate burden) and ≥ 8 points (severe burden). The standardised mean difference (SMD) was calculated for baseline characteristics and outcome variables. SMD was used to calculate the difference between the means of two groups divided by their standard deviations. Values below 0.1 (10%) were considered inconsequential.

A multivariable logistic regression analysis was conducted to estimate the association between the ACCI (low, moderate or severe burden) and CFS ≥ 5, with documented treatment limitations being the outcome variable expressed as an odds ratio (OR) with a 95% confidence interval (95% CI). The model was adjusted for age, sex and care setting. To evaluate if the inclusion of only valid treatment limitations changed the outcome of the multivariable logistic regression analysis, a sub-group analysis was performed. Non-valid treatment limitations were reclassified as “No treatment limitation” and the multivariable logistic regression was repeated with Treatment limitation as the dependent variable and care setting, age, sex, ACCI and CFS as the independent variables.

A p-value < 0.05 (two-sided) was considered statistically significant. All analyses were performed using the R statistical software [30].

## Results

### Study population and characteristics

A total of 500 older adult patients, 218 men and 282 women, from in-patient and out-patient care settings (MSTC, HHS) were included. The gender distribution of patients in medical departments, surgery departments and MSTC was fairly even, whereas female gender dominated in orthopaedic units and HHS (Tables 1 and 2).

**Table 1:** Demographic and clinical characteristics of patients aged 80 and over across medicine, orthopaedic and surgery wards

	Medicine *n* (%)	Orthopaedic *n* (%)	Surgery *n* (%)	*p*-value
Patients	100	100	100	
Age = 80–89 years	69 (69.0)	63 (63.0)	80 (80.0)	0.03
Age = ≥ 90 years	31 (31)	37 (37)	20 (20.0)	
Male	46 (46.0)	40 (40.0)	47 (47.0)	0.56
Female	54 (54.0)	60 (60)	53 (53.0)	
*Diagnosis at admission*				< 0.001
Cardio-pulmonary disease	44 (44.0)	2 (2.0)	2 (2.0)	
Fracture	1 (1.0)	59 (59.0)	2 (2.0)	
Gastrointestinal	2 (2.0)	0 (0.0)	41 (41.0)	
Infection	22 (22.0)	8 (8.0)	13 (13.0)	
Malignancy	3 (3.0)	0 (0.0)	15 (15.0)	
Musculoskeletal pain	1 (1.0)	13 (13.0)	0 (0.0)	
Neurology	10 (10.0)	0 (0.0)	0 (0.0)	
Non-specific complaint	11 (11.0)	4 (4.0)	4 (4.0)	
Psychiatric/Cognitive	4 (4.0)	0 (0.0)	0 (0.0)	
Surgery	2 (2.0)	5 (5.0)	11 (11.0)	
Trauma	0 (0.0)	9 (9.0)	12 (12.0)	
*Clinical Frailty Scale*				0.72
CFS 1–4: Not frail	14 (14.0)	12 (12.0)	16 (16.0)	
CFS 5–9: Frail	86 (86.0)	88 (88.0)	84 (84.0)	
*Age-combined Charlson Comorbidity Index*				0.08
0–4 points	0 (0.0)	3 (3.0)	3 (3.0)	
5–7 points	50 (50.0)	56 (56.0)	40 (40.0)	
≥ 8 points	50 (50.0)	41 (41.0)	57 (57.0)	
*Level of care*				
Treatment limitation	54 (54.0)	33 (33.0)	42 (42.0)	0.01
DNAR	53 (53.0)	33 (33.0)	41 (41.0)	0.01
DNI	7 (7.0)	0 (0)	5 (5.0)	0.03
Not for ICU	28 (28.0)	13 (13.0)	13 (13.0)	0.006
DNAR decision, current care	43 (43.0)	24 (24.0)	33 (33.0)	0.02
DNAR decision, reviewed/confirmed	6 (6.0)	2 (2.0)	2 (2.0)	0.19
DNAR decision, non-valid *Participation by patients and relatives*	4 (4.0)	7 (7.0)	6 (6.0)	0.42
Patient informed/participation	10 (10.0)	7 (7.0)	13 (13.0)	0.37
Only relatives informed	8 (8.0)	1 (1.0)	5 (5.0)	0.06
Information to patients or relatives	18 (18.0)	8 (8.0)	17 (17.0)	0.08

*DNAR* Do not attempt resuscitation, *DNI * Do not intubate, *ICU * Intensive care unit

**Table 2:** Demographic and clinical characteristics of patients aged 80 and over in home health service (HHS) and municipal Short-Term care (MSTC)

	HHS *n* (%)	MSTC *n* (%)	*p*-value	SMD
Patients	100	100		
Age = 80–89 years	62 (62.0)	61 (61.0)	1.00	0.021
Age = ≥ 90 years	38 (38.0)	39 (39.0)		
Sex = Male	39 (39.0)	46 (46.0)	0.39	0.142
Sex = Female	61 (61.0)	54 (54.0)		
*Clinical Frailty Scale*			1.00	0.082
CFS 1–4: Not frail	1 (1.0)	2 (2.0)		
CFS 5–9: Frail	99 (99.0)	98 (98.0)		
*Age-combined Charlson Comorbidity Index*			0.31	0.218
0–4 points	1 (1.0)	3 (3.0)		
5–7 points	52 (52.0)	43 (43.0)		
≥ 8 points *Level of care*	47 (47.0)	54 (54.0)		
Treatment limitation	56 (56.0)	59 (59.0)	0.89	0.040
DNAR	56 (56.0)	59 (59.0)	0.89	0.040
DNAR decision, current care	30 (30.0)	17 (17.0)	0.07	0.280
DNAR decision, reviewed/confirmed	9 (9.0)	10 (10.0)	1.00	0.034
DNAR decision, non-valid *Participation by patients and relatives*	17 (17.0)	32 (32.0)	0.02	0.354
Patient informed/participation	21 (21.0)	20 (20.0)	1.00	0.025
Only relatives informed	5 (5.0)	10 (10.0)	0.28	0.190
Information to patients or relatives *Other care level limitations*	26 (26.0)	30 (30.0)	0.64	0.089
Hospital care if deteriorated (physician decision)	89 (89.0)	93 (93.0)	0.46	0.140
Hospital care only in case of trauma/fracture	8 (8.0)	3 (3.0)	0.22	0.221
No hospital care (patient decision)	2 (2.0)	4 (4.0)	0.68	0.117

*DNAR * Do not attempt resuscitation, *SMD * Standardized mean difference

More patients in the orthopaedic department, MSTC and HHS were ≥ 90 years compared to those in the surgical and medical departments (Tables 1 and 2). Among those receiving HHS, 58% were cared for in their private residences and 42% in nursing homes. Of the patients, 335 were aged 80–89 years and 165 ≥ 90 years (Table 3).

**Table 3:** Comparison of demographic and clinical characteristics between patients receiving full care and those with treatment limitations

	Full care *n* (%)	Treatment limitation *n* (%)	*p*-value	SMD
Patients	256	244		
*Ward/Institution*			0.001	0.400
Hospital ward - internal medicine	46 (18.0)	54 (22.1)		
Hospital ward - orthopaedics	67 (26.2)	33 (13.5)		
Hospital ward - surgery	58 (22.7)	42 (17.2)		
HHS	44 (17.2)	56 (23.0)		
MSTC	41 (16.0)	59 (24.2)		
Age 80–89 years	209 (81.6)	126 (51.6)	< 0.001	0.671
Age ≥ 90 years	47 (18.4)	118 (48.4)		
Male	106 (41.4)	112 (45.9)	0.36	0.091
Female	150 (58.6)	132 (54.1)		
*Clinical Frailty Scale*			< 0.001	0.588
CFS 1–4: Not frail	43 (16.8)	2 (0.8)		
CFS 5–9: Frail	213 (83.2)	242 (99.2)		
*Age-combined Charlson Comorbidity Index*			< 0.001	0.381
0–4 points	9 (3.5)	1 (0.4)		
5–7 points	140 (54.7)	101 (41.4)		
≥ 8 points *Level of care*	107 (41.8)	142 (58.2)		
DNAR	0 (0.0)	242 (99.2)		
DNI	0 (0.0)	12 (4.9)		
Not for ICU	0 (0.0)	54 (22.1)		
DNAR decision, current care	0 (0.0)	147 (60.7)		
DNAR decision, reviewed/confirmed	0 (0.0)	29 (11.9)		
DNAR decision, non-valid *Participation by patients and relatives*	0 (0.0)	66 (27.3)		
Patient informed/participation	0 (0.0)	71 (29.1)		
Only relatives informed	0 (0.0)	29 (29.0)		
Information to patients or relatives	0 (0.0)	100 (41.0)		

*HHS * Home Health Service, *MSTC * Municipal Short-term Care, *DNAR * Do not attempt resuscitation, *DNI * Do not intubate, *ICU * Intensive care unit, *SMD *Standardized mean difference

Among hospital patients, 82% had received care for ≤ 7 days at the time of the review, 14% for 8–14 days and 4% ≥15 days. Of patients in MSTC, 67% had been registered for ≤ 30 days, 28% for 1–3 months and 5% for ≥ 3 months. Among HHS patients, a third had been registered for ≤ 1 year, a third for 1–3 years and a third for ≥ 3 years.

### Diagnoses on admission

The most common diagnoses on admission in medical departments were cardio-pulmonary disease (44%) and infection (22%), in orthopaedic departments fractures (59%) and musculoskeletal pain (13%) and in surgical units, gastro-intestinal diseases (41%) and malignancies (15%) (Table 1). In MSTC the reason for admission was non-specific complaint (general deterioration), care/rehabilitation following hospitalisation due to fractures, infection, cognitive decline, trauma and malignancy. In HHS the reasons for care were help with drug dispensing and everyday care.

### Comorbidity

**Fig. 1:**
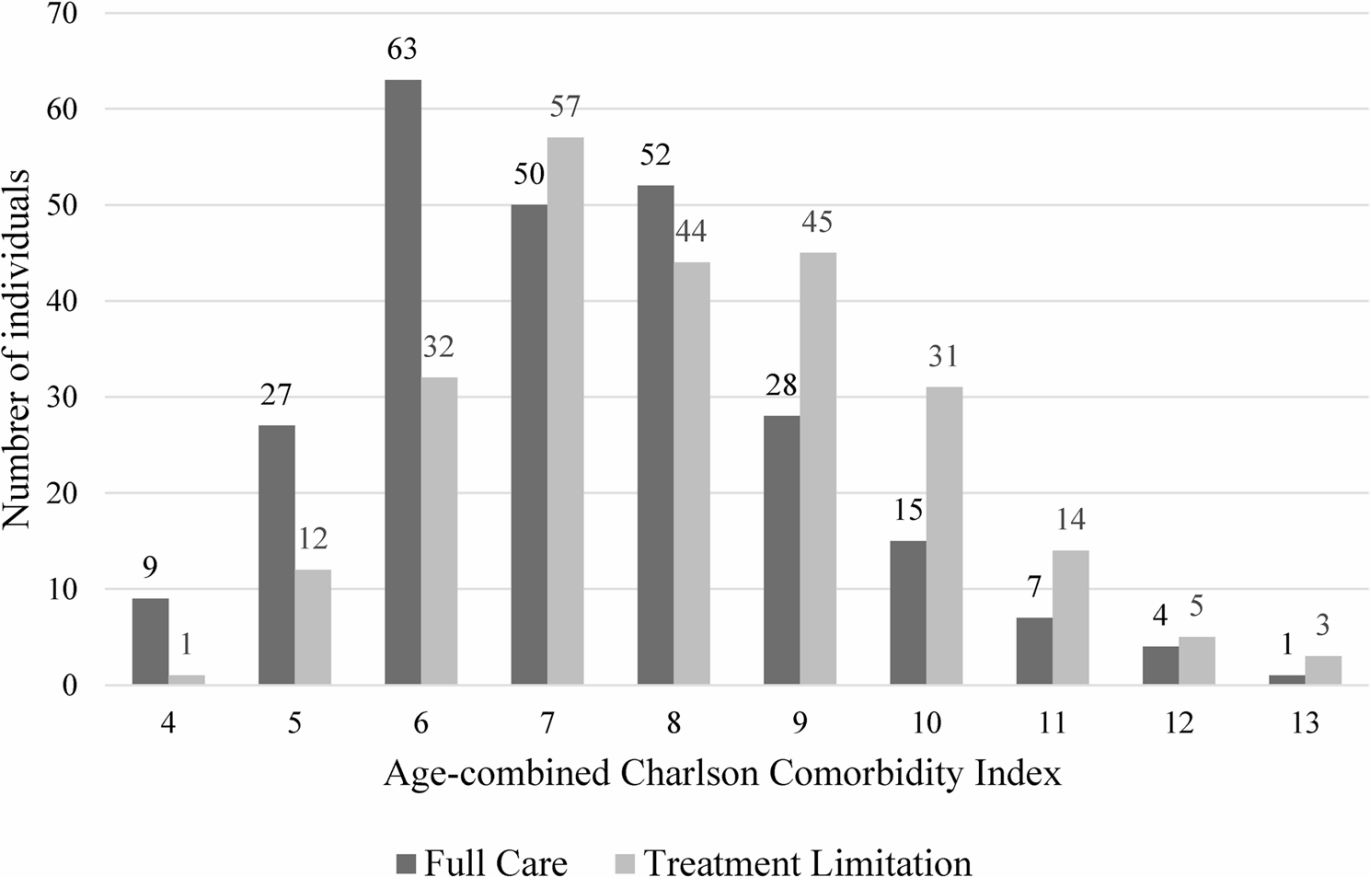
Comparison of full care and treatment limitation by Aged-combined Charlson Comorbidity Index distribution

The most common comorbidity diagnoses in hospitals were peripheral vascular disease, malignancy, and heart failure (Supplement 1). Peripheral vascular disease was also the most frequent comorbidity in the MSTC and the HHS, followed by cerebrovascular disease, dementia and diabetes (Supplement 2). Many patients had ≥ 2 comorbidity diagnoses. Ten of the 500 patients had no comorbidity, with ACCI 4 points confined to those aged 80–89 years. In total, 241 patients had ACCI 5–7 points, while 249 had ACCI ≥ 8 points (Table 3). Figure 1 shows the distribution of patients and ACCI-scores 4–13.

### Frailty and DNAR-decisions

**Fig. 2:**
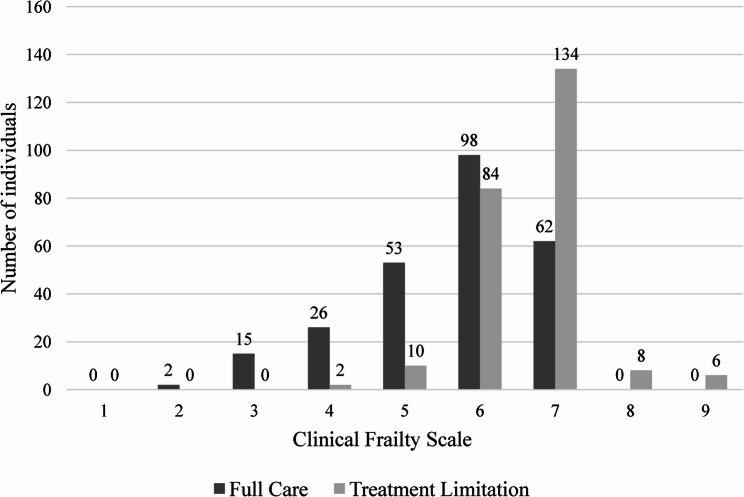
Comparison of full care and treatment limitation by Clinical Frailty Scale distribution

Of the 500 patients, only 45 were rated as non-frail, two of whom had a DNAR-decision (Fig. 2). In total, 455/500 (91%) were rated as frail (frailty ≥ 5), the largest proportion was rated as 6 or 7 on the CFS (Fig. 2).

**Fig. 3:**
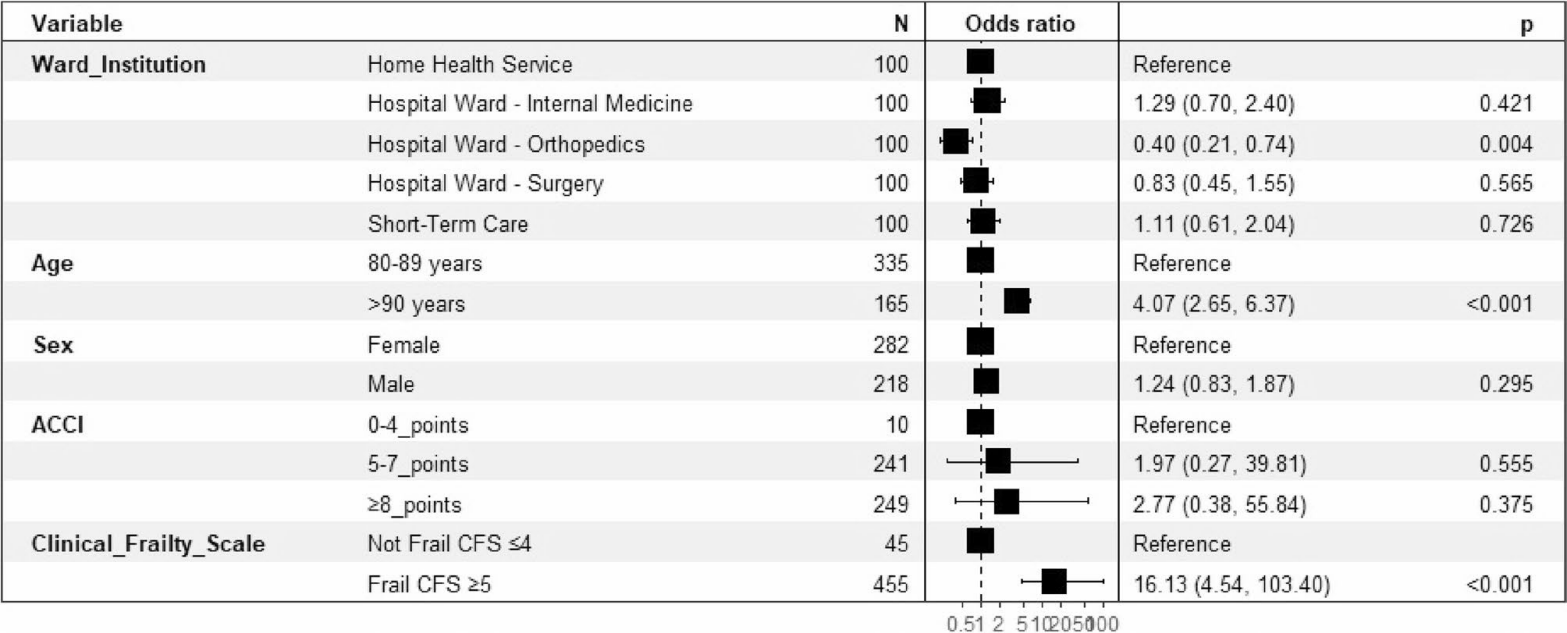
Forest plot summarizing the multivariable logistic regression model for prediction of treatment limitations in patients aged 80 and over. The X-axis shows the odds ratios. The corresponding error bars illustrate the 95% confidence intervals. *ACCI * Age-combined Charlson Comorbidity Index, *CFS * Clinical Frailty Scale

Of those with frailty rating 6, 84/182 (46%) had a DNAR-decision. Among those with frailty rating 7, 134/196 (68%) had such a decision. All patients with an 8 or 9 frailty rating had a DNAR-decision (Fig. 2). In the multivariable analysis, both age ≥ 90 years (OR 4.07, 95% CI 2.56–6.37) and CFS ≥ 5 (OR 16.13, 95% CI 4.54–103.40) was significantly associated with a treatment limitation which is illustrated in Fig. 3. Patients cared for at the orthopaedic hospital ward were independently less likely to receive a treatment limitation (OR 0.40, 95% CI 0.21–0.74).

### ACCI and DNAR-decisions

In 10/500 patients (2%) with no comorbidity on the ICD 10, one had a DNAR-decision (Fig. 1). Of those with ACCI 5–7 points, 101/241 (42%) had a DNAR-decision and among those with ACCI ≥ 8 the figure was 142/249 (57%) (Fig. 1). The OR did not show any significant difference in terms of a DNAR-decision in those with ACCI ≥ 8 points (Fig. 3). In the univariable analysis, ACCI ≥ 8 points were significant for a DNAR-decision (p = < 0.001), as well as age ≥ 90 years (p = < 0.001), (Table 3).

### Valid and non-valid DNAR-decisions

Out of the 300 hospital patients, 127 had a DNAR-decision documented in their medical records, 100 of which were made during the current hospitalisation, while 10 from the previous care form were actively reviewed/confirmed. Thus, 110/300 patients (37%) had a valid DNAR-decision pertaining to the current form of care, while 17/127 decisions (13%) were not reviewed and confirmed (hence non-valid) (Table 1). Among the 200 patients in HHS and MSTC, 115 had DNAR-decisions documented in their records, whereas 47 decisions made in the current and 19 from the previous care form had been reviewed/confirmed. Therefore, 66/200 patients (33%) had valid DNAR-decisions, while 49/115 decisions (43%) were non-valid (i.e. not reviewed/confirmed) (Table 2). The total number of patients with valid DNAR-decisions was 176/500 (35%). Of the 244 patients with a treatment limitation, 12 had a Do-not-intubate (DNI) decision of which two had unrestricted CPR (Table 3). A sub-group analysis including only valid treatment limitations (multivariable logistic regression, see supplement 3) showed similar results as the original multivariable logistic regression.

### Time to DNAR-decisions in the current form of care

In the 3 participating hospital clinics, most of the DNAR-decisions (58/100) had been made on admission. A further 30 decisions were made within 1–3 days and 12 after more than 3 days. In HHS, 5 DNAR-decisions had been made ≤ 1 month after registration, 11within one year and 14 ≥ 1 year after registration. In MSTC, 11 DNAR-decisions were made ≤ 10 days after admission, 6 within 11 days to 1 month.

### Other care level and treatment limitations

Among hospitalised patients the most common care level limitation was being deemed unable to cope with intensive care in case of deterioration. In the 3 hospital clinics, 54 patients had a Not for ICU-decision, while 12 had a DNI-decision (Table 1).

Patients in MSTC and HHS had other care level limitations; 18 patients were not supposed to be hospitalised in case of deterioration and instead receive palliative care in their current location. Of these, 6 had decided that they did not want to be transferred to hospital in the event of deterioration (Table 3). A total of 11 patients from the two above-mentioned care forms should only be transferred to hospital to treat a trauma/fracture.

### Participation by patients and relatives

The records of 71/242 patients (29%) with a DNAR-decision revealed that a physician had informed them about the decision during a dialogue. In the case of patients who could not participate, 29 relatives (12%) had been informed. In 38/242 records (16%) both the patient and a relative had received information. In 142/242 patients (59%), neither patient nor relative were told about the DNAR-decision (Table 3). Among 256 patients with unrestricted interventions, no discussion with the physician in charge was documented. Thus, of the total population, 71/500 (14%) received information about CPR.

## Discussion

This study, encompassing the medical records of 500 patients ≥ 80 years receiving various forms of care, shows that 50% had a severe burden of ACCI (≥ 8 points) and that 91% were frail (CFS ≥ 5). In total, 35% had valid DNAR-decisions. Frailty was significantly associated with the presence of a DNAR-decision. No significant relationship was found between ACCI ≥ 8 points and treatment limitation in the multivariable analysis. Nearly a third (29%) of patients with a DNAR-decision had discussed it with a physician. For the 256 patients with full care, no documentation stating that they had participated in the decision could be found, thus only 14% of 500 patients had received information or been offered discussions about CPR. Some of the patients in HHS and MSTC also had other care limitations, such as no hospitalisation in the event of deterioration and hospitalisation only in cases of trauma and fracture.

Like other studies [1]– [2], our study revealed fewer documented decisions than expected regarding treatment limitations in patients ≥ 80 years. In a study from 2017, only 16% had a documented DNAR-decision within 24 h of hospital admission [1]. Such decisions should be documented early in the care process to avoid burdensome and futile resuscitation attempts [1]. In the case of hospital patients in our study, 58/100 CPR-decisions had been documented on admission and a further 30 within 1–3 days. This may indicate enhanced awareness among hospital staff related to medical-ethical CPR-decisions. In HHS and MSTC, decisions were taken after a considerably longer time or documented by the previous caregiver. A substantially larger number of DNAR-decisions (43%) had not been actively reviewed/confirmed when compared with the hospital population (13%). Unconfirmed DNAR decisions create moral distress, uncertainty, and make it difficult for staff to act in an emergency, as they are obliged to contact the physician concerned to obtain confirmation of the decision. Although the inclusion or exclusion of non-valid treatment limitations did not change the over-all conclusion of the study (that frailty was associated with treatment limitations), the results raise important questions. Our results suggest that many physicians perceived non-valid treatment limitations as valid. It is possible that this was due to a lack of knowledge of current guidelines or a perceived lack of time. HHS care plans should be regularly reviewed to meet patients’ care needs, as they are expected to deteriorate over time [31]. This is also true of MSTC, where patients’ health often deteriorates. Consequently, patients should be asked about their wishes and the outcome documented early in the care process [2].

In our study, 37% of the hospital population had valid DNAR-decisions. A study conducted in hospitals in the Netherlands demonstrated that DNAR was documented in 66.4% of patients over 80 years, in 47.6% of those with ACCI ≥ 5 and in 54.7% of those with ACCI ≥ 8 [32]. These figures are higher than among our hospital population. Patients with a moderate ACCI burden (5–7 points) have little chance of surviving cardiac arrest in hospital, while among those with a severe burden (ACCI ≥ 8) the chance is minimal [3]. Considering that almost half of the patients in our study had ACCI 5–7 and the other half ACCI ≥ 8, more patients could be expected to have had a valid DNAR-decision. The number of patients with a DNAR-decision would probably have been larger, had more been asked whether they wished to receive full CPR in the event of cardiac arrest. A study showed that one of the factors affecting DNAR/DNI-decisions is whether the patient participated, as patients themselves are more willing to refrain from CPR and mechanical ventilation than when a third party makes the decision [33]. Medical-ethical guidelines [14–16] describe the patient’s right to participation, including the possibility of declining life-sustaining interventions. The most common wish among hospital patients with severe chronic comorbidity, malignancy or multimorbidity who had a DNAR-decision, was to refrain from CPR [18].

Of the 200 patients in HHS and MSTC, 33% had valid DNAR-decisions. These figures agree with those in another study, where 30% of patients in sub-acute care had DNAR-decisions, which percentage increased after hospitalisation [2]. Such a tendency was also evident in our study, where 37% of hospitalised patients had valid DNAR-decisions. However, we observed a clearer difference between hospital clinics, with medical departments having 49% valid DNAR-decisions, compared to orthopaedic (26%) and surgical departments (35%). In another study, more internal medicine patients also had DNAR-decisions compared to surgical specialities [32]. In our study, common diagnoses on admission to the medical clinic were deteriorating cardiopulmonary disease (44%) and acute infection (22%). This probably increased the likelihood of DNAR-decisions, compared to those in the surgical and orthopaedic departments who had less life-threatening conditions. Older patients who had DNAR-decisions in conjunction with surgery had a significantly higher burden of morbidity and mortality compared to those with no such decision [34]. Thus, older patients at risk of adverse events in relation to surgery must receive the opportunity to participate in discussions about the content of care and acceptable risks. Furthermore, the limitations Not for ICU and DNI were more common among medical patients compared to orthopaedic and surgical ones. Other studies have also reported a significantly higher incidence of such treatment limitations in medical units [35].

Among the 200 HHS and MSTC patients in our study, other care limitations were reported, such as: no hospitalisation in the event of deterioration (9%) and hospitalisation only in cases of trauma/fracture (5%). Some of these patients were in a late phase of life, as 7% had 8 or 9 on the CFS (very severely frail or terminally ill). In another Swedish study examining the records of 367 nursing home patients, 77% had advanced care plans in which patients and/or their family members had participated [31]. Care limitations regarding hospitalisation were found in 72%. It was also reported that 81% had DNAR-decisions, which is considerably more than the 39% of HHS patients in our study. Patients and family members had also been far more involved in the care planning compared to our findings [31].

The prevalence of frailty was high in our study with 91% of all 500 patients having CSF ≥ 5, of whom 42% had CFS 7–9. Research has revealed that the chance of survival after cardiac arrest in the presence of frailty is small, with figures as low as 1.8% in those with CFS 6–9 [36]. Another study found that hospital mortality was 84% in those with CFS 7–9 [37]. In our study, frailty was significant (p = < 0.001) for a DNAR-decision. This could be interpreted as an increased awareness among healthcare staff in various forms of care that frail patients have a poor prognosis for survival after cardiac arrest. The results also suggest that clinicians more often base DNAR decisions on functional prognosis (CFS) rather than diagnostic disease burden. One study reported that frail patients who received CPR in hospital survived with similar neurological functions as before the arrest but only one in five survived for 3 years. They also had increased risk of depression and poorer general health compared to non-frail patients [38]. In our study, DNAR decisions made during the current episode of care were most common on medical wards, despite similar levels of frailty being observed in orthopaedic and surgical wards. This suggests that a sudden deterioration in functional prognosis in elderly patients contributes to the initiation of DNAR decisions. This becomes particularly evident on medical wards, were acute conditions such as severe infections, respiratory complications, or general prognostic decline necessitate consideration of treatment limitations. In contrast, on orthopaedic or surgical wards, acute life-threatening organ failure is less common, which means that DNAR decisions are not prompted to the same extent.

In our study, participation through information/discussion was found in 24% of hospitalised patients with a DNAR-decision. This corresponds with other Swedish studies, where 26% [39] and 28% [18] of hospitalised patients on general wards and only 17% of patients in the ICU [40] had received information about DNAR-decisions. Studies from other European countries have demonstrated that 65.9% of those aged ≥ 80 had participated in discussions about CPR [32] and that the corresponding figure for patients in medical departments was 43.6% [41]. The lower proportion of hospitalised patients in our study who had discussed a DNAR-decision can be due to the situation on admission, when their condition was often acute, making such a discussion impossible. In HHS and MSTC, where there should be time for dialogue, only 20% of patients with a DNAR-decision had received information. One explanation might be inadequate routines and/or lack of knowledge about medical-ethical guidelines [9, 14–16]. In the 500 records reviewed in our study, the figure for conversation/participation in CPR-decisions was a mere 14%. The results may indicate a lack of awareness among clinicians that failing to offer elderly patients with a high disease burden and significant frailty the opportunity to discuss treatment limitations violates both ethical standards and patients’ legal rights. Organisational factors that may contribute include a lack of continuity among staff and increasing shorter hospital stays at the units studied, which may make it more difficult to detect deterioration in individual patients. Another Swedish study has also highlighted poor adherence to Swedish legal and ethical decision-making principles, emphasising the need for a more systematic approach to documentation and dialogue with patients, their relatives, and healthcare colleagues [19].

### Strengths and limitations

The inclusion of real-world documentation across different healthcare settings provides valuable insights into the gap between guidelines and clinical practice. Another strength of this study is its focus on frailty as a predictor of treatment limitation decisions, using real-world clinical data and validated tools like the CFS and ACCI.

A limitation of this study was the use of different nursing documentation systems, for out-patient care. Therefore, information about patients’ current status and frailty level had to be searched for in different sources. Care plans were not standardised, making it time-consuming to identify relevant information. It is possible that discussions with patients regarding DNAR may have taken place and been agreed upon but were not documented in the medical record. However, such cases are likely to represent only a very small proportion of the included records and are therefore not considered to have influenced the results. Furthermore, the study was performed in one healthcare region in Sweden, which limits generalizability, as the results might have differed had the study been carried out in another region.

## Conclusion

We found fewer documented treatment limitation decisions than expected for older and frail patients with a high burden of comorbidity. Considerable deficiencies were revealed related to decision-making or actively reviewing decisions. This was particularly true in HHS and MSTC. Clinicians should use the CFS to guide them in discussions with patients about their future care. It could be integrated as a tool within DNAR protocols. Frailty assessment opens the possibility of engaging in structured conversations with the patient and their relatives about the goals of care, at a much earlier stage than during an emergency. It is essential to increase the knowledge about ACCI to assist in CPR decision-making. There is a gap between guideline recommendations and how they are implemented in clinical practice. Far too few patients have the opportunity to receive information and participate in dialogue about DNAR-decisions or are informed about their right to decline CPR in the event of a cardiac arrest. One way of dealing with the ethical dilemmas experienced by healthcare staff may be to shift the focus from a specific DNAR-decision to formulating an individualised care plan together with the patient. This study highlights the need for increased awareness and knowledge of ethical guidelines concerning CPR. Improved communication and documentation are essential prerequisites for greater adherence to medical-ethical guidelines. This would strengthen patients’ legal rights and their opportunity to participate in decision-making.

## Supplementary Information

Supplementary Material 1.

Supplementary Material 2.

## Data Availability

All data generated or analysed during this study are included in this published article [and its supplementary information files].

## Abbreviations

ACCI: Age-combined Charlson Comorbidity Index
ANOVA: Analysis of variance
CFS: Clinical Frailty Scale
CI: Confidence interval
CPR: Cardiopulmonary resuscitation
DNAR: Do not attempt resuscitation
DNI: Do not intubate
HHS: Home Health Services
ICD: International Classification of Diseases
ICU: Intensive care unit
MSTC: Municipal Short-term Care
n: Number of patients/observations
OR: Odds ratio
SD: Standard deviation
SMD: Standardized mean difference
