# Supplementary material for: Treatment limitations and participation in elderly patients – the gap between medical-ethical guidelines and clinical practice: a cross sectional-study from Sweden
Source: BMC Geriatr. 2025 Nov 4;25:841. doi: 10.1186/s12877-025-06552-x (PMC12584302; doi:10.1186/s12877-025-06552-x)
Supplement: Supplementary file 1 — Supplementary Material 1. [file 12877_2025_6552_MOESM1_ESM.docx]

**Supplementary material**

Supplementary Table 1: Comorbidities assessed according to ICD-10, in medicine, orthopaedic and surgery wards.

|  |  | Medicine  *n* (%) | Orthopaedic  *n* (%) | Surgery  *n* (%) | p-value |
| --- | --- | --- | --- | --- | --- |
|  | Total number of patients | 100 | 100 | 100 |  |
|  | Acute myocardial infarction | 18 (18.0) | 15 (15.0) | 18 (18.0) | 0.88 |
|  | Heart failure | 34 (34.0) | 16 (16.0) | 22 (22.0) | 0.01 |
|  | Peripheral vascular disease | 92 (92.0) | 83 (83.0) | 79 (79.0) | 0.03 |
|  | Cerebrovascular disease | 23 (23.0) | 22 (22.0) | 22 (22.0) | 0.98 |
|  | Dementia | 6 (6.0) | 13 (13.0) | 8 (8.0) | 0.20 |
|  | Chronic obstructive pulmonary disease | 14 (14.0) | 7 (7.0) | 8 (8.0) | 0.19 |
|  | Chronic pulmonary disease, other | 4 (4.0) | 12 (12.0) | 6 (6.0) | 0.08 |
|  | Rheumatoid arthritis | 4 (4.0) | 5 (5.0) | 1 (1.0) | 0.26 |
|  | Hemiplegia | 4 (4.0) | 3 (3.0) | 0 (0.0) | 0.15 |
|  | Diabetes without complications | 17 (17.0) | 9 (9.0) | 15 (15.0) | 0.23 |
|  | Diabetes with complications | 3 (3.0) | 4 (4.0) | 5 (5.0) | 0.77 |
|  | Chronic kidney disease | 24 (24.0) | 16 (16.0) | 18 (18.0) | 0.33 |
|  | Liver disease mild | 0 (0.0) | 0 (0.0) | 2 (2.0) | 0.13 |
|  | Liver disease severe | 1 (1.0) | 0 (0.0) | 0 (0.0) | 0.37 |
|  | Ulcus | 8 (8.0) | 0 (0.0) | 6 (6.0) | 0.02 |
|  | Cancer | 26 (26.0) | 25 (25.0) | 31 (31.0) | 0.59 |
|  | Metastatic cancer | 4 (4.0) | 5 (5.0) | 22 (22.0) | <0.001 |

**Supplementary material**

Supplementary Table 2: Comorbidities assessed according to ICD-10, in Home Health Services (HHS) and Municipal Short-Term Care (MSTC).

|  |  | HHS  *n* (%) | MSTC  *n* (%) | p-value |
| --- | --- | --- | --- | --- |
|  | Total number of patients | 100 | 100 |  |
|  | Acute myocardial infarction | 14 (14.0) | 10 (10.0) | 0.51 |
|  | Heart failure | 21 (21.0) | 18 (18.0) | 0.72 |
|  | Peripheral vascular disease | 83 (83.0) | 88 (88.0) | 0.42 |
|  | Cerebrovascular disease | 43 (43.0) | 29 (29.0) | 0.06 |
|  | Dementia | 31 (31.0) | 33 (33.0) | 0.88 |
|  | Chronic obstructive pulmonary disease | 7 (7.0) | 5 (7.0) | 0.77 |
|  | Chronic pulmonary disease, other | 7 (7.0) | 5 (5.0) | 0.77 |
|  | Rheumatoid arthritis | 3 (3.0) | 2 (2.0) | 1.00 |
|  | Hemiplegia | 10 (10.0) | 8 (8.0) | 0.81 |
|  | Diabetes without complications | 15 (15.0) | 12 (12.0) | 0.68 |
|  | Diabetes with complications | 12 (12.0) | 16 (16.0) | 0.54 |
|  | Chronic kidney disease | 11 (11.0) | 14 (14.0) | 0.67 |
|  | Liver disease mild | 0 (0.0) | 1 (1.0) | 1.00 |
|  | Liver disease severe | 2 (2.0) | 0 (0.0) | 0.48 |
|  | Ulcus | 2 (2.0) | 1 (1.0) | 1.00 |
|  | Cancer | 15 (15.0) | 18 (18.0) | 0.70 |
|  | Metastatic cancer | 3 (3.0) | 10 (10.0) | 0.08 |
