# Supplementary material for: Treatment limitations and participation in elderly patients – the gap between medical-ethical guidelines and clinical practice: a cross sectional-study from Sweden
Source: BMC Geriatr. 2025 Nov 4;25:841. doi: 10.1186/s12877-025-06552-x (PMC12584302; doi:10.1186/s12877-025-06552-x)
Supplement: Supplementary file 2 — Supplementary Material 2. [file 12877_2025_6552_MOESM2_ESM.docx]

**Supplementary material**


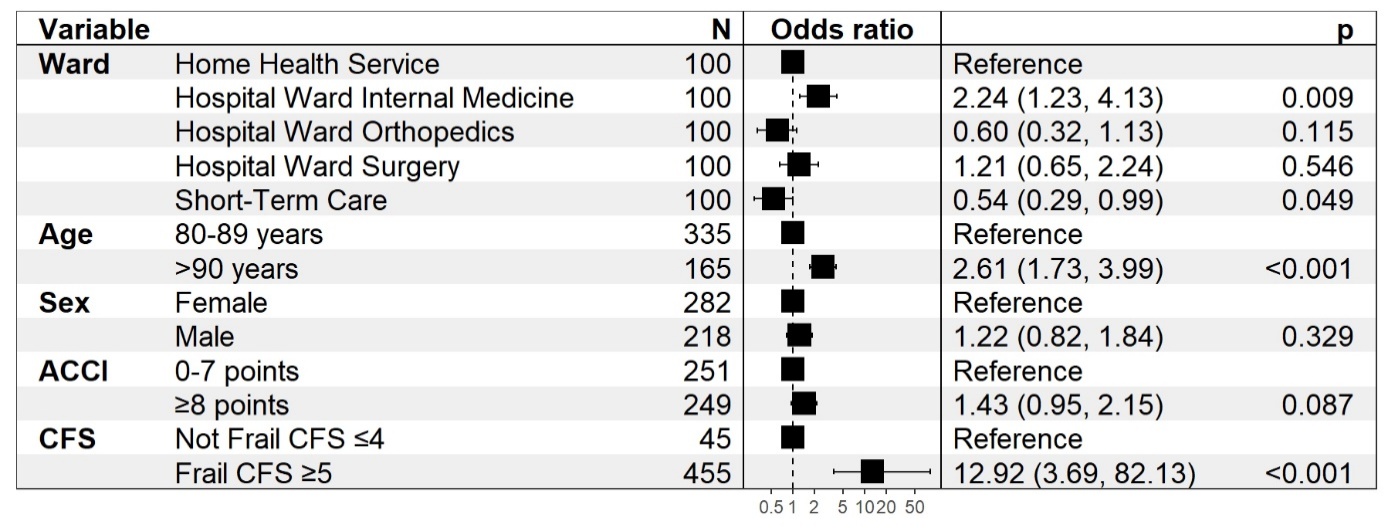


ACCI = Age-combined Charlson Comorbidity Index; CFS = Clinical Frailty Scale

**Figure 4.** Subgroup analysis: The Forest plot shows the results of a multivariable logistic regression analysis of factors associated with the establishment of a treatment limitation. The analysis includes only cases with treatment limitations that were validated and established in accordance with Swedish legislation and ethical guidelines.
